# RT-qPCR analyses on the osteogenic differentiation from human iPS cells: an investigation of reference genes

**DOI:** 10.1038/s41598-020-68752-2

**Published:** 2020-07-16

**Authors:** Kensuke Okamura, Yusuke Inagaki, Takeshi K. Matsui, Masaya Matsubayashi, Tomoya Komeda, Munehiro Ogawa, Eiichiro Mori, Yasuhito Tanaka

**Affiliations:** 1 Department of Orthopaedic Surgery, Nara Medical University, Kashihara, Japan; 2 Department of Future Basic Medicine, Nara Medical University, Kashihara, Japan

**Keywords:** Molecular biology, Stem cells

## Abstract

Reverse transcription quantitative PCR (RT-qPCR) is used to quantify gene expression and require standardization with reference genes. We sought to identify the reference genes best suited for experiments that induce osteogenic differentiation from human induced pluripotent stem cells. They were cultured in an undifferentiated maintenance medium and after confluence, further cultured in an osteogenic differentiation medium for 28 days. RT-qPCR was performed on undifferentiation markers, osteoblast and osteocyte differentiation markers, and reference gene candidates. The expression stability of each reference gene candidate was ranked using four algorithms. General rankings identified TATA box binding protein in the first place, followed by transferrin receptor, ribosomal protein large P0, and finally, beta-2-microglobulin, which was revealed as the least stable. Interestingly, universally used GAPDH and ACTB were found to be unsuitable. Our findings strongly suggest a need to evaluate the expression stability of reference gene candidates for each experiment.

## Introduction

Quantitative real-time PCR (qPCR) was developed in the 1990s^1^ and is now widely used in various fields as a tool for the accurate and easy detection as well as quantification of target nucleic acid samples. In particular, reverse transcription quantitative PCR (RT-qPCR) is regarded as an indispensable experimental technique for the analysis of gene expression by quantifying RNA.

RT-qPCR is used to measure the amplification of an exponentially amplified region and quantify its initial template cDNA, that is, mRNA. Accurate assessments require the correction of inter sample variations, particularly of factors such as the total amount and quality of RNA, reverse transcription efficiency, and PCR efficiency. To perform this, internal control genes are selected as reference genes, which are quantified along with the target gene. Standardization is performed by calculating their expression ratios. The “housekeeping genes”, those with constant expression levels in the tissues or cells to be analyzed, are generally used as internal control genes; however, their expression levels may vary with each tissue and each cell. Fluctuations have been reported depending on the conditions such as the stages of development^2^. The Minimum Information for Publication of Quantitative Real-Time PCR Experiments (MIQE), a set of guidelines compiled to ensure the reproducibility of qPCR, stipulate that reference genes suited for each cell, tissue, and experimental design be selected from numerous candidates so as to ensure reliability of normalisation^3^. It is evident hence, that the selection of suitable reference genes, along with the provision of data demonstrating their expression stability, is becoming an indispensable part of quantitative analysis of gene expression using RT-qPCR.

To date, research has mainly focused on the osteogenic potential of mesenchymal stem cells (MSCs)^4,5^. However, since Takahashi and Yamanaka’s^6^ reports on human induced pluripotent stem (iPS) cells, there have been high expectations for the use of human iPS cells in areas such as regenerative medicine^7^ and the development of drugs for rare diseases^8^. Cell culture experiments have focused on hypoxic environments as the original physiological state inside living tissues^9–11^. We reported that when rat MSCs cultured in a hypoxic environment were exposed to a normoxic environment, it enhanced their osteogenic potential^12^. Based on these results, we expect an examination of the osteogenic differentiation cultures of iPS cells under hypoxic conditions would significantly contribute to osteochondral regenerative medicine and our understanding of the pathology of musculoskeletal diseases such as knee osteoarthritis. Nevertheless, only a few studies have thoroughly verified the expression stability of reference genes used during RT-qPCR for osteogenic differentiation experiments using stem cells, especially iPS cells. Therefore, in the present study we compared multiple internal control genes to identify the most stable reference genes for osteogenic differentiation experiments using RT-qPCR on human iPS cells under various oxygen concentrations.

## Results

### Tissue staining

**Figure 1:**
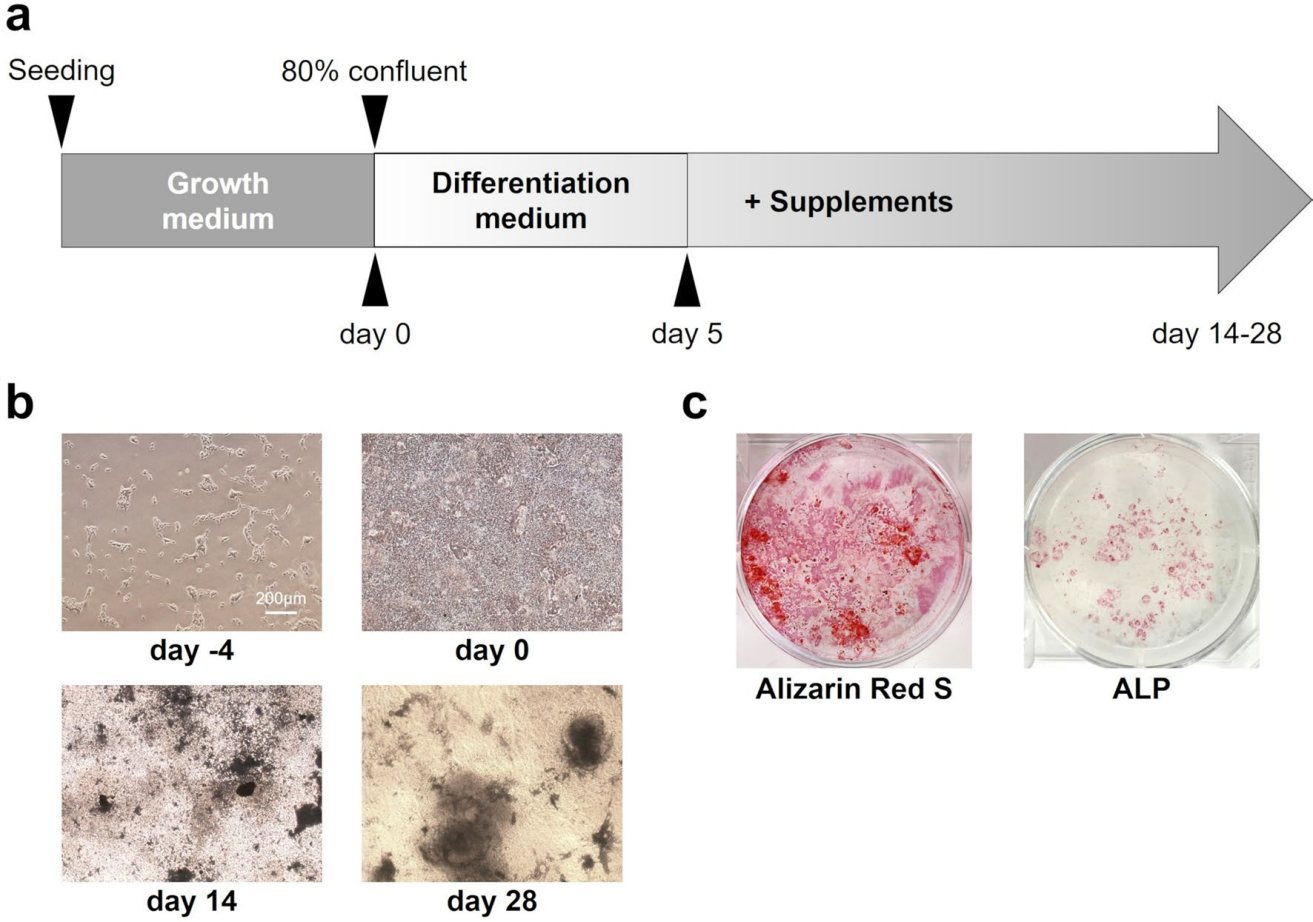
Osteogenic differentiation in human induced pluripotent stem (iPS) cells. (**a**) Schema of the osteogenic differentiation protocol. (**b**) Microscopic images on day − 4, 0, 14 and 28 showed mineralization yield. (**c**) Mineralization on day 28 with Alizarin red S and alkaline phosphatase (ALP) staining results in six-well culture plates.

Continuous induction of osteogenic differentiation (Fig. 1a) resulted in areas that revealed calcification (Fig. 1b). Samples cultured on day 28 were stained to verify this. The results presented areas positive for Alizarin Red S throughout the wells, with certain regions exhibiting ALP activity (Fig. 1c).

### Comparison of reference gene candidate stability by RT-qPCR

**Figure 2:**
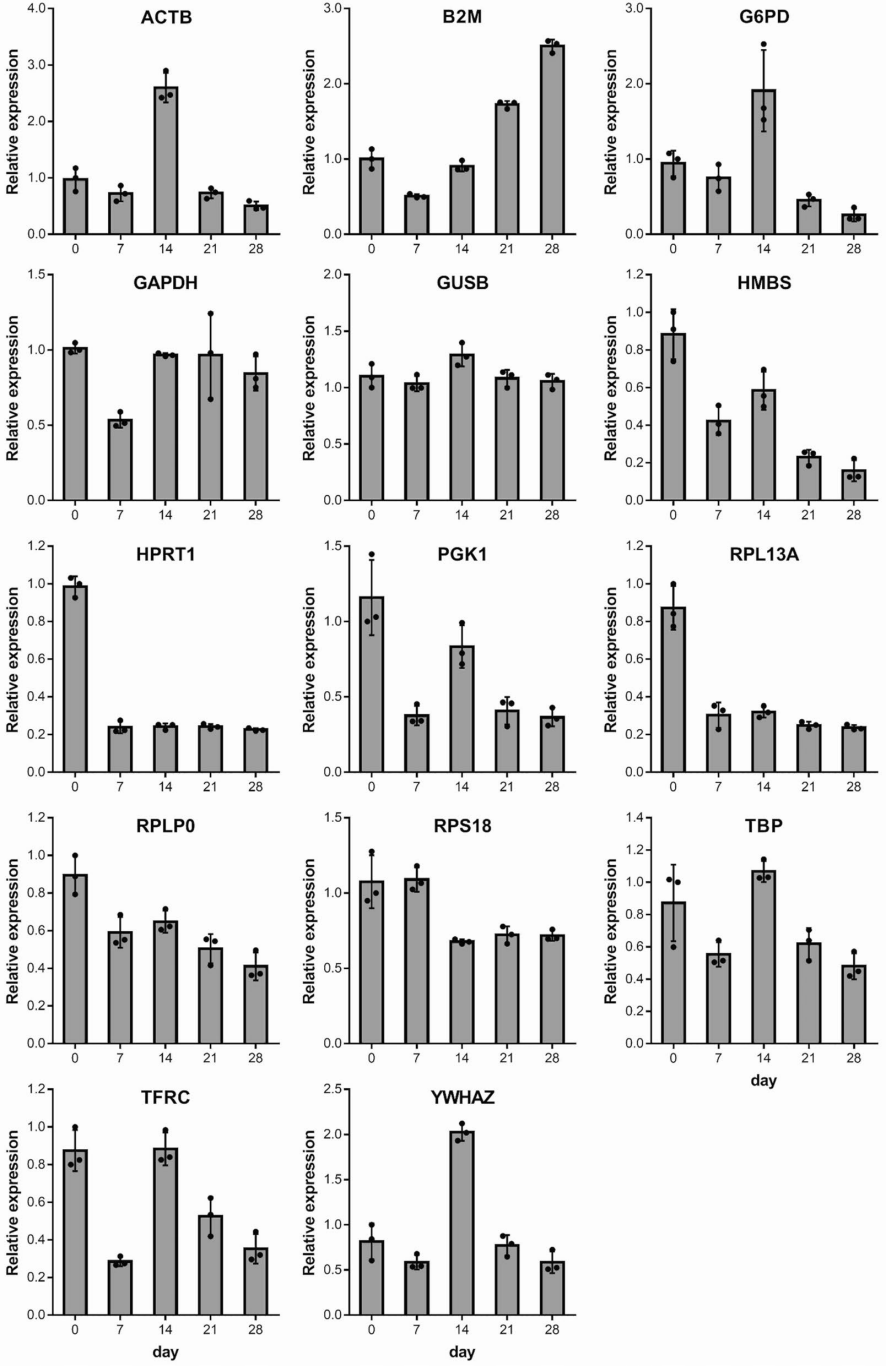
Expression of reference genes in iPSCs during osteogenic differentiation. The values are represented as the mean ± S.D. from three independent samples.

RT-qPCR was performed to compare the expression stability of internal control genes listed in Table 1 on samples from three wells on days 0, 7, 14, 21, and 28. The resulting threshold cycle (Ct) values (Fig. 2) and PCR efficiency values from the validation data of each primer were used in each method to calculate the results listed in Table 2. None of the samples were excluded as invalid while verifying RNA purity with absorbance measurements, and all Ct values were < 35.

**Table 1:** Primers of candidate reference genes.

Symbol	Name	Function	Assay ID (Bio-Rad)	Amplicon length (bp)	Product efficiency
ACTB	actin, beta	Cytoskeletal structural protein	qHsaCED0036269	62	2.03
B2M	beta-2-microglobulin	Beta chain of MHC class I	qHsaCID0015347	123	1.98
G6PD	glucose-6-phosphate dehydrogenase	Maintain the level of NADPH	qHsaCED0001353	137	2.05
GAPDH	glyceraldehyde-3-phosphate dehydrogenase	Glycolysis and gluconeogenesis	qHsaCED0038674	117	1.97
GUSB	glucuronidase, beta	Carbohydrate metabolic process	qHsaCID0011706	79	2.03
HMBS	hydroxymethylbilane synthase	Heme biosynthetic process	qHsaCID0038839	137	1.98
HPRT1	hypoxanthine phosphoribosyltransferase 1	Purine salvage pathway	qHsaCID0016375	90	2.05
PGK1	phosphoglycerate kinase 1	Glycolysis and gluconeogenesis	qHsaCED0003721	74	1.81
RPL13A	ribosomal protein L13a	Component of the 60S ribosomal subunit	qHsaCED0020417	118	1.99
RPLP0	ribosomal protein, large, P0	Component of the 60S ribosomal subunit	qHsaCED0038653	63	1.95
RPS18	ribosomal protein S18	Component of the 40S ribosomal subunit	qHsaCED0037454	67	1.94
TBP	TATA box binding protein	Ttranscription factor binding	qHsaCID0007122	120	2.01
TFRC	transferrin receptor	Transferrin transport	qHsaCID0022106	102	1.96
YWHAZ	14-3-3 protein, zeta polypeptide	Major regulator of apoptotic pathways	qHsaCID0013897	175	1.96

**Table 2:** Expression stability and ranking of reference genes, evaluated based on ΔCt method, BestKeeper, NormFinder, and geNorm.

Reference gene	ΔCt		BestKeeper					NormFinder		geNorm		Comprehensive ranking	
	Mean SD	Rank	SD	Rank	Coeff. of corr	Rank	General rank	Stability value	Rank	M value	Rank	Geometric mean of ranks	General rank
ACTB	0.739	9	0.650	9	0.777*	7	10	0.678	13	0.591	10	10.400	11
B2M	1.185	14	0.697	11	0.001	14	14	0.891	14	0.751	14	14.000	14
G6PD	0.838	13	0.818	13	0.816*	6	12	0.500	12	0.674	13	12.490	13
GAPDH	0.654	5	0.337	3	0.610*	11	7	0.168	6	0.404	4	5.384	5
GUSB	0.651	4	0.110	1	0.596*	12	2	0.127	3	0.386	3	2.913	4
HMBS	0.788	11	0.827	14	0.876*	4	9	0.162	5	0.620	11	8.590	9
HPRT1	0.805	12	0.635	8	0.642*	10	13	0.290	9	0.647	12	11.393	12
PGK1	0.699	7	0.762	12	0.930*	2	5	0.199	7	0.476	6	6.192	6
RPL13A	0.698	6	0.544	6	0.751*	8	8	0.225	8	0.535	8	7.445	7
RPLP0	0.568	2	0.342	4	0.869*	5	4	0.128	4	0.339	1	2.378	3
RPS18	0.746	10	0.307	2	0.106	13	6	0.323	10	0.512	7	8.050	8
TBP	0.560	1	0.416	5	0.906*	3	3	0.068	1	0.339	1	1.316	1
TFRC	0.634	3	0.651	10	0.936*	1	1	0.068	1	0.443	5	1.968	2
YWHAZ	0.718	8	0.567	7	0.721*	9	10	0.440	11	0.564	9	9.434	10

*P < 0.05.

**Figure 3:**
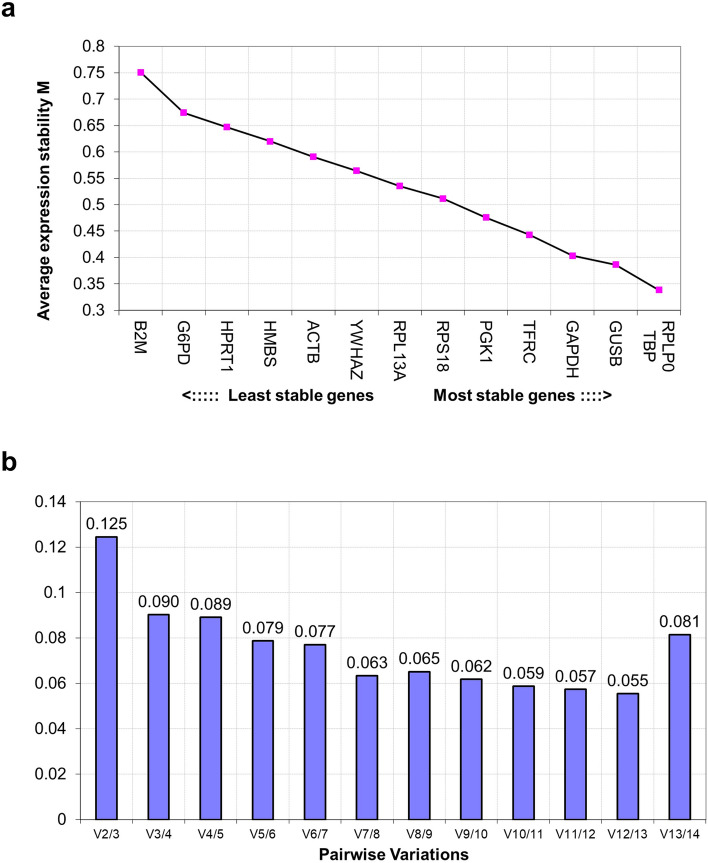
geNorm analysis results. (**a**) Ranking based on average expression stability M of internal control genes. (**b**) Optimal number of control genes required for reliable normalization calculated by the pairwise variation (V_n/n + 1_).

First, the ΔCT method revealed that the gene with the lowest mean SD was TBP, and that with the highest value was B2M, which was the only one with a mean SD > 1. Based on BestKeeper threshold of SD = 1, no candidate genes were excluded. GUSB and HMBS had the lowest and highest SDs, respectively. In addition, r value was the highest and lowest for TFRC and B2M, respectively. The general ranking calculated from these two rankings revealed that TFRC had the highest stability, while that of B2M was the lowest. Using NormFinder, all the iPS cell samples in this study that underwent osteogenic differentiation were treated as a single group. Three samples from each day were used to analyze a total of 15 samples. The genes with the lowest stability values were TBP and TFRC, whereas B2M had the highest value. Figure 3 depicts two graphs created using geNorm. The pair with the lowest M value was RPLP0 and TBP with threshold values of less than 0.5, whereas B2M presented the highest value (Fig. 3a). In addition, V_2/3_–V_13/14_ were all below 0.15 (Fig. 3b). These results indicate that valid assessments can be performed with the combination of RPLP0 and TBP. While these methods found several candidates with high stability, including TBP and TFRC, all the methods demonstrated the low stability of B2M.

A general ranking was calculated using the geometric means of the rankings in each of the four methods (Table 2). The results indicate that TBP is the gene with the most stable expression and that B2M is the lowest.

### Expression of target genes using RT-qPCR

**Figure 4:**
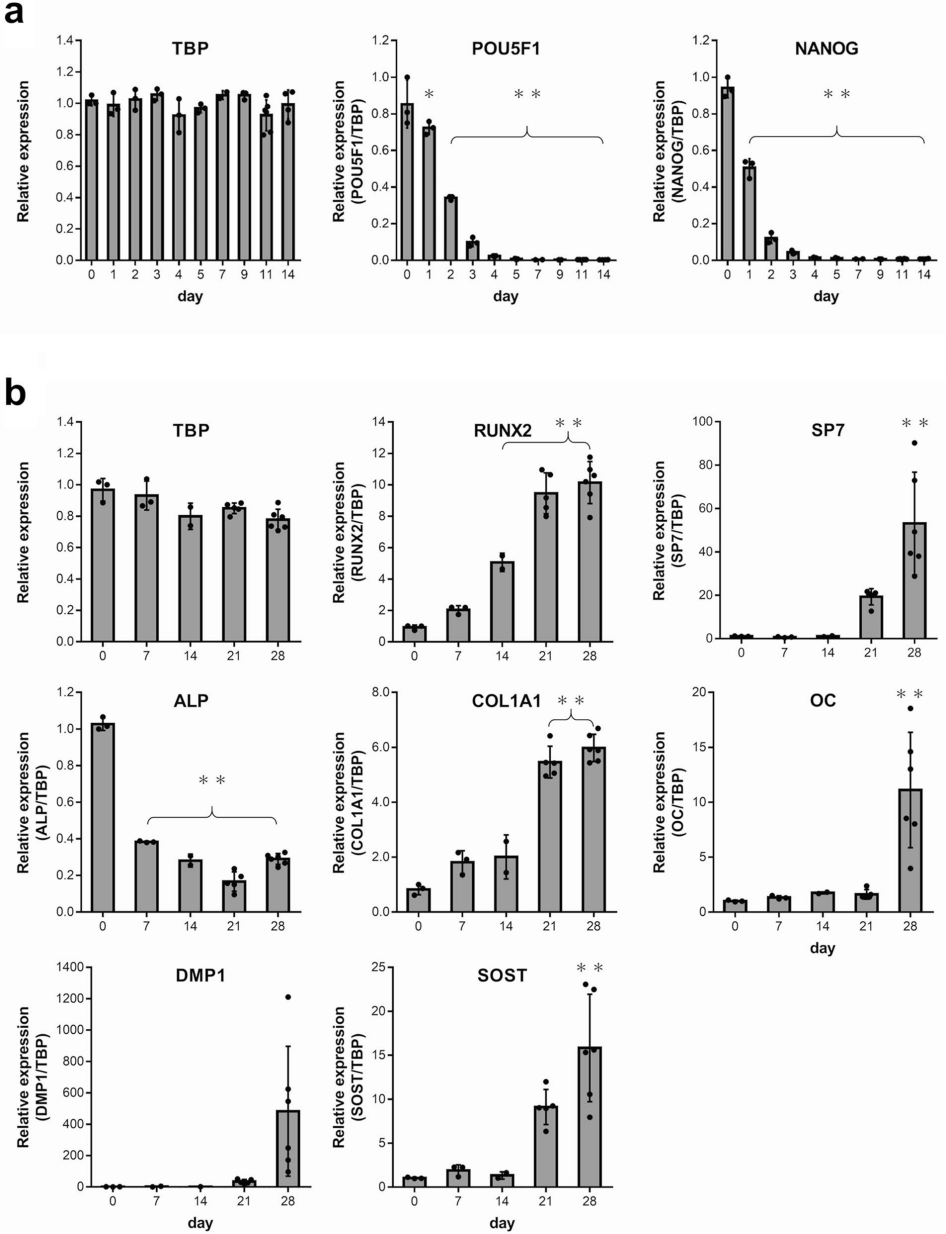
Expression of undifferentiation (**a**) and osteogenic marker (**b**) genes standardized by TBP in iPSCs during osteogenic differentiation. The values are represented as the mean ± S.D. from two-six independent samples. One-way analysis of variance (ANOVA) with Bonferroni correction for multiple comparisons was applied. *P < 0.05, **P < 0.01 versus day 0.

Using TBP as the reference gene based on the results of the aforementioned analysis, RT-qPCR was performed to evaluate the expression of each target gene in the osteogenic differentiation process using the primers listed in Table 3. Like the process with internal control genes, samples from days 0, 7, 14, 21, and 28 were used. To consider the variation in differentiation rates between samples on days 21 and 28, six wells were sampled on these days and three wells were sampled on the other days. For the undifferentiation markers, sampling was performed on days 0, 1, 2, 3, 4, 5, 7, 9, 11, and 14, given that gene expression declines early in the culture period. Due to the variation in differentiation rates between samples, six wells were sampled on days 11 and 14, and three wells were sampled on the other days. Relative expression levels for each gene were calculated using the 2^−∆∆Ct^ method by employing the Ct values obtained by RT-qPCR (Fig. 4). A verification of RNA purity using absorbance measurements revealed that A_260_/A_230_ was greater than 1.8 in the following samples: one sample on day 7 and two samples on day 14 in the undifferentiation marker experiment; one sample on day 14 and one sample on day 21 in the experiment conducted for the other markers. These samples were deemed as invalid and excluded from the analysis. As the Ct values of DMP1 exceeded 35 in one sample each on days 7 and 14, these samples were also excluded as they were deemed invalid.

**Table 3:** Primers of target genes.

Assay	Symbol	Name	Function	Assay ID
PrimePCR SYBR Green Assay (Bio-Rad)	TBP	TATA box binding protein	Transcription factor binding	qHsaCID0007122
	POU5F1	POU class 5 homeobox 1	Marker for undifferentiated cells	qHsaCED0038334
	NANOG	Nanog homeobox	Marker for undifferentiated cells	qHsaCED0043394
	ALP	Alkaline phosphatase, liver/bone/kidney	Matrix mineralization	qHsaCED0045991
	OC	Osteocalcin	Calcium-binding protein hormone	qHsaCED0038437
	DMP1	Dentin matrix acidic phosphoprotein 1	Extracellular matrix mineralization	qHsaCID0015301
	SOST	Sclerostin	Produced primarily by the osteocyte	qHsaCED0046506
Taqman Gene Expression Assay (Applied Biosystems)	RUNX2	Runt related transcription factor 2	Key transcription factor for osteoblast differentiation	Hs00231692_m1
	SP7	Sp7 transcription factor	Transcription factor for osteoblast differentiation	Hs01866874_s1
	COL1A1	Collagen type I alpha 1	Fibrillar collagen	Hs00164004_m1

Figure 4a depicts the expression of TBP as a reference gene and the changes in the relative expression of undifferentiation markers during osteogenic differentiation up to day 14. TBP expression did not vary markedly over the entire period. The expression of both undifferentiation markers, POU5F1 and NANOG, declined significantly from day 1 until day 14. This suggests that during the osteogenic differentiation process in this study, human iPS cells lost their undifferentiated nature and further differentiated into a certain cell type.

Figure 4b illustrates the expression of TBP as a reference gene and the changes in the relative expression of osteoblast and osteocyte differentiation markers during osteogenic differentiation up to day 28. TBP expression did not vary significantly over the entire period. Expression of the transcription factors, RUNX2 and SP7, and the proteins, COL1A1 and OC, which are osteoblast differentiation markers, exhibited increasing trends starting from day 0. These results indicate that inducing osteogenic differentiation promoted osteoblast differentiation. The protein ALP is not only an osteoblast differentiation marker but also an undifferentiation marker. Its expression level was high on day 0 when the cells were undifferentiated. It then declined over time before it exhibited an increasing trend again on day 28 due to osteoblast differentiation. The protein DMP1 is an osteocyte differentiation marker that is expressed in young osteocytes and the protein SOST is expressed in mature bone cells. Both exhibited a marked increase in expression on day 28. Despite the large variations in DMP1 expression between samples on day 28, osteocyte differentiation is assumed to have progressed at least during the second half of the osteogenic differentiation period.

We also standardized the results of RT-qPCR using only GAPDH (Supplemental Fig. 1), which is described as one of the most commonly used method in existing studies^13,14^. When the focus was on the presence of significant differences, results were generally similar. However, we found the relative expression of GAPDH tended to be higher in the second half of the osteogenic differentiation period compared to TBP, and the relative expression of the target genes tended to be relatively low.

These results indicate that human iPS cells used in the present study differentiated into osteoblasts and osteocytes by inducing osteogenic differentiation. They also provide evidence that assessment of the expression of internal control genes, which was the objective of this study, was performed during the induction of osteogenic differentiation in human iPS cells.

## Discussion

Presently, we conduct osteogenic differentiation experiments using human iPS cells, and RT-qPCR is used as the primary method to quantify gene expression. This speaks to the importance of the accuracy of RT-qPCR and the need to appropriately evaluate the quality of the extracted RNA and select reagents and reference genes.

In the present study, we initially examined the RNA extraction from calcified samples. First, the absorbance of the RNA samples was measured to calculate A_260_/A_230_ and A_260_/A_280_ ratios. High RNA purity was observed when A_260_/A_230_ > 1.8 and A_260_/A_280_ > 2.0. Denatured agarose gel electrophoresis was also performed on the RNA samples to calculate the ribosomal RNA ratio 28S/18S and determine whether it was close to 2:1. Assessing the RNA quality of several extraction reagents revealed that TRIzol had the highest probability of clearing the RNA purity criteria with 28S/18S close to 2:1. This reagent was found to yield maximum RNA from samples of the same size. Based on these results, we determined that TRIzol is the most suitable for extracting RNA in osteogenic differentiation experiments and devised a method of using RNeasy Micro Kit to recover RNA after extraction.

Reference Genes H96 comprises preset control wells for verifying genomic DNA contamination, PCR efficiency, RNA quality, and reverse transcription efficiency. We used this plate in a preliminary experiment for the present study and found a case that did not meet the standard for reverse transcription efficiency during control. This prompted us to compare multiple cDNA synthesis kits using reagents used in control wells for the reverse transcription efficiency of Reference Genes H96. The iScript Advanced cDNA Synthesis Kit had the highest reverse transcription efficiency and was adopted for cDNA synthesis.

RNA concentrations were calculated from A_260_ measured with a spectrophotometer and cDNA concentrations were calculated from RNA concentration. We recognize that their lack of accuracy may affect the assessment of gene expression levels and is a limitation of this study. However, these concentrations must be standardized by suitable reference genes and hence in the present study, we did not require further accuracy.

After the aforementioned verification process, we verified suitable reference genes, which was the aim of the present study. Standardizing the expression of target genes using reference genes is an essential means of correcting variations between samples in areas such as total RNA, reverse transcription efficiency, and PCR efficiency. The MIQE guidelines state that suitable reference genes should be selected for each tissue, cell, and experimental design^3^. However, an existing study on osteogenic differentiation using human embryonic stem (ES) cells, MSCs, and iPS cells was found to use customarily used genes such as GAPDH, ACTB, and 18S rRNA^13^. The use of GAPDH is particularly common, although no basis has been provided for its selection^14^. Only limited studies have attempted to identify optimal reference genes, and as far as we could find, none were identified for human iPS cells.

To verify the optimal reference genes, the present study used the most common and reliable methods: the ΔCt method, BestKeeper, NormFinder, and geNorm. The ΔCt method is a pairwise comparison and details on the other three algorithms have been cited in the MIQE guidelines^3^. Reference genes verification results based on these algorithms have been reported in several studies for various cells and differentiation processes^15–23^. For example, Robledo et al.^16^ and Augustyniak et al.^17^ performed verifications using the four algorithms we used in the present study. Jacob et al.^15^ suggested that verifications should be performed with at least three different algorithms. They found that more than 60% of the published studies on RT-qPCR use two algorithms, NormFinder and geNorm, whereas only 2.6% use all four algorithms. This was based on their review of studies published between 2009 and 2012. Although we expect recent studies to apply more algorithms, Jacob et al.’s finding highlighted the fact that only a few studies have conducted thorough reference gene verification.

Due to the weighting of SD and r values, Bestkeeper may be the least user-friendly out of the four algorithms. As no clear ranking method is provided, some studies have chosen not to use this method^18^. In the present study, after excluding items with SD > 1 as invalid, we calculated a general ranking by measuring the geometric mean ranking of SD and r value. A web-based tool called RefFinder (https://www.leonxie.com/referencegene.php) automatically executes BestKeeper, NormFinder, and geNorm algorithms when Ct values are entered, and calculates the stability rankings based on each algorithm, as well as a general ranking. Although many studies have used the verification results of RefFinder^15,19–21^, we did not adopt it in the present study because; (1) it does not take into account PCR efficiency, which can affect PCR results^21^; (2) its BestKeeper results only include SD but not r; and (3) the method used to determine the general ranking is not stipulated.

Of the 14 preset candidate genes in Reference Genes H96, we found B2M to be the most unsuitable reference gene. This finding was consistent across all the algorithms and our results indicate that B2M has high fluctuations compared to the other candidates. Consistent with Jacob et al.^15^ and Vandesompele et al.’s^24^ recommendation to evaluate combinations of three reference genes, our results found TBP, TFRC, and RPLP0 to be suitable reference genes. The details of our findings are presented in Table 2.

However, in the present study we obtained these results only on the osteogenic differentiation of human iPS cells under normoxic conditions. We plan to perform further experiments under hypoxic conditions and with the exclusion of candidate genes that fluctuate with changes in oxygen concentration. GAPDH is a typical example of such genes. Among the three recommended genes, TFRC is involved in the hypoxic response, and as it is presumed to fluctuate depending on the oxygen concentration, its exclusion is highly recommended^25^. Hence, if only one reference gene was to be used, it should be TBP. If a combination of genes was to be used, geNorm results indicate that the standard should be TBP and RPLP0. The present study found TBP to have the highest stability, a finding which is consistent with that of Rauh et al.^22^ They found TBP to be the most stable reference gene in NormFinder and geNorm in three-dimensional osteogenic differentiation culture experiments of human bone marrow derived MSC. Consistent with their results, we also found TFRC to be the second most stable reference gene. However, instead of TBP or TFRC, their investigation of reference genes in a two-dimensional culture experiment identified completely different genes as the most suitable. Li et al.^23^ found that TBP to be one of the least stable genes and B2M as one of the most stable in osteogenic differentiation and adipose differentiation culture experiments of human bone marrow-derived and fetal tissue-derived MSCs. Their results appear to be contradictory to our findings. However, this issue of reversed stability ranking of genes has been raised by Rauh et al.^22^ and suggests that the selection of reference genes suited to each cell, tissue, and experimental design requires an investigation of the expression stability indicated by reference gene candidates in each experiment.

We would like to highlight that the commonly used GAPDH was not highly ranked; its position was 5 out of 14, whereas ACTB was positioned at 11. Our results indicate that commonly used reference genes such as GAPDH and ACTB were unsuitable in the current experiment and that using them may have led to incorrect interpretations of gene expression levels. This is consistent with De Jonge et al.’s^26^ analysis of data from public samples of 13,629 microarrays performed in various tissues and conditions; they found that of the 13,037 genes, none of the commonly used reference genes—ACTB, GAPDH, HPRT1, B2M—were ranked in the top 50 for stability.

Our study standardized the results of RT-qPCR on target genes in the osteogenic differentiation process using only TBP, which was used to evaluate the expression level of each gene. In this study, POU5F1 and NANOG were used as undifferentiation markers and RUNX2, SP7, ALP, COL1A1, and OC were used as osteoblast differentiation markers. These genes were adopted because they were commonly used in studies^27–30^ reporting similar osteogenic differentiation experiments on human iPS cells. We considered osteocyte differentiation markers to be necessary as well. We used DMP1, which is expressed in relatively young osteocytes, and SOST, which is a marker of mature osteocytes. We found that the levels of undifferentiation markers declined markedly within 2 weeks, indicating a loss of undifferentiation, whereas those of the osteoblast and osteocyte differentiation markers increased within 4 weeks. This indicates that iPS cells differentiate into osteoblasts and osteocytes. This observation was also supported by results from two types of staining. We were able to achieve the main purpose of this study, that is to verify the reference genes in an osteogenic differentiation experiment on human iPS cells. When the reference gene was changed from TBP to GAPDH, there was a slight difference in the trend of relative expression of the target genes. Although it did not appear as a clear difference in this study, there is a risk that even a slight difference in the trend of relative expression could lead to errors in the assessment of target gene expression levels.

The stage of differentiation that the human iPS cells were at each time in the culture period of our experiments was not revealed. This is another limitation of this experiment. However, given that the purpose of this study was to validate the reference genes which can be used appropriately throughout the entire culture period, we decided that it was not necessary to verify the differentiation stage.

As we continue to develop our osteogenic differentiation experiments using iPS cells, it will be necessary to verify the transition of the differentiation stage by quantification of target proteins and other factors. We believe that this will allow us to verify suitable reference genes at each differentiation stage. In addition, the verification of more genes that have not been included in the reference gene candidates so far, will allow room for the identification of more suitable reference genes. We hope that these verification data will allow to perform more accurate RT-qPCR.

## Materials and methods

### Human iPS cell line and induction of osteogenic differentiation

We used the commercially available Cellartis Human iPS Cell Line 12 (Takara Bio Inc., Kusatsu, Japan) as the human iPS Cell line. This was seeded on wells coated with the Matrigel basement membrane matrix for feeder-free culturing (Corning Inc., Corning, NY, USA) and then cultured in the undifferentiated maintenance medium Essential 8 (E8; Thermo Fisher Scientific Inc., Waltham, MA, USA). The medium was changed daily and after reaching confluence (about 80%), the cells were detached using Versene (Thermo Fisher Scientific Inc.) for 12 min in 37 °C and replated. This passage procedure was repeated.

Cells obtained by cloning in this manner were used in the osteogenic differentiation experiment (Fig. 1a). Confluent cells (defined as Day 0) were switched to Dulbecco’s modified Eagle’s medium (DMEM; Nacalai Tesque Inc.) containing fetal bovine serum (FBS, 15%; GE Healthcare Life Sciences, Logan, UT, USA), 1% nonessential amino acids (Sigma-Aldrich Inc., St. Louis, MO, USA), 1% antibiotic–antimycotic mixed stock solution (Nacalai Tesque Inc., Kyoto, Japan), and 100 μM β-mercaptoethanol (Nacalai Tesque Inc.). This medium was changed daily. From day 5,10 mM β-glycerophosphate (Sigma-Aldrich), 50 μg/ml l-ascorbic acid (Wako Pure Chemical Ind., Osaka, Japan), 10 nM dexamethasone (Sigma-Aldrich), and 50 nM 1,25-(OH)2 vitamin D_3_ (Wako Pure Chemical Ind.) were added to the above-mentioned medium to induce osteogenic differentiation and the culture was changed once every two days.

### Tissue staining

For the staining assessments, the human iPS cells that underwent osteogenic differentiation were fixed with 95% ethanol. To evaluate Ca deposition, they were stained with Alizarin Red S (Nacalai Tesque Inc.). To assess ALP activity, they were stained with Naphthol AS-MX phosphate disodium salt (Sigma-Aldrich) and Fast Red Violet LB Salt (Nacalai Tesque Inc.) in 56 mM 2-amino-2-methyl-1,3-propanediol buffer (Wako Pure Chemical Ind.) adjusted to pH 9.9.

### Gene expression analysis

For total RNA extraction, TRIzol (Invitrogen Life Technologies, Carlsbad, CA, USA) was added and left to stand at room temperature for 10 min. The resulting solution was transferred to a QIA shredder spin column (Qiagen Inc., Valencia, CA, USA) and centrifuged at 15,000 rpm for 2 min at room temperature. The resulting filtrate was transferred to a new 1.5 ml microtube, 200 μl of chloroform was added, mixed well, and allowed to stand at room temperature for 5 min. This was centrifuged at 15,000 rpm for 15 min at 4 °C before the upper aqueous layer was transferred to a new 1.5 ml microtube, and the same amount of 70% ethanol was added and mixed well. This sample was then transferred to RNeasy Micro Kit spin column (Qiagen Inc.) and total RNA was recovered according to the procedure described in the product’s instruction manual. Next, DNase treatment was performed to prevent genomic DNA contamination. The resulting solution was diluted 20-fold with Tris–EDTA buffer (pH 8.0) (Nippon Gene, Toyama, Japan), and then analyzed with a Ultrospec 3,000 spectrophotometer (Biochrom Ltd., Cambridge, England) at 230, 260, and 280 nm to measure the absorbance, based on which the purity of RNA was confirmed and its concentration was calculated. RNA template (1 μg) was prepared and reverse transcription reaction was performed with iScript Advanced cDNA Synthesis Kit (Bio-Rad Laboratories, Hercules, CA, USA) according to the procedure described in the product’s instruction manual. The StepOnePlus Real-Time PCR System (Applied Biosystems, Foster City, CA, USA) was used for RT-qPCR.

Reference Genes H96 (Bio-Rad Laboratories) was used in RT-qPCR to compare the expression levels of several internal control genes that were candidate reference genes. Reference Genes H96 is a predesigned plate with wells containing primers for 14 types of validated human internal control genes (Table 1), which can be used as control assays for evaluating genomic DNA contamination, PCR efficiency, RNA quality, and reverse transcription efficiency. A reaction solution of 20 µl comprising 10 μl 2 × Sso Advanced universal SYBR Green Supermix (Bio-Rad), 1 μl cDNA template (25 ng), and 9 μl sterile distilled water was added per sample in each well. For the reaction conditions, initial activation was performed at 95 °C for 2 min, followed by 40 cycles of thermal denaturation at 95 °C for 5 s and then annealing and elongation at 60 °C for 30 s, and eventually the melting curve was analyzed.

The internal control genes selected based on the data analysis described in the following manner were used as the target genes in RT-qPCR to analyze the expression of undifferentiation, osteoblast differentiation, and osteocyte differentiation markers (Table 3). PrimePCR SYBR Green Assay (Bio-Rad) or Taqman Gene Expression Assay (Applied Biosystems) was used as the primer. For the former, 5 μl of 2 × SsoAdvanced universal SYBR Green Supermix, 0.5 μl of 20 × SYBR Green Assay, 1 μl (25 ng) of cDNA template, and 3.5 μl of sterile distilled water were used as the reaction solution, for a total of 10 μl per sample dispensed in each well. The reaction conditions were the same for Reference Genes H96. For the latter, 10 μl of 2 × Taqman Fast Universal PCR Master Mix, 1 μl of 20 × Gene Expression Assay, 2 μl (50 ng) of cDNA template, and 7 μl of sterile distilled water were used as the reaction solution, for a total of 20 µl per sample dispensed in each well. For the reaction conditions, initial activation was performed at 95 °C for 20 s, followed by 40 cycles of thermal denaturation at 95 °C for 1 s, and annealing and elongation at 60 °C for 20 s.

### Data analysis

To select suitable reference genes for osteogenic differentiation experiments in human iPS cells, Ct values, PCR efficiency, and relative expression values based on the internal control genes obtained using RT-qPCR were used in the following methods for analysis.

#### ΔCt method

Silver et al.^31^ reported the ΔCt method for comparing the stability of candidate genes based on pairwise comparison. A pair is created with candidate gene A and another candidate gene B. Thereafter, the difference in their Ct values in the same sample is calculated to obtain ΔCt. The standard deviation (SD) of ΔCt in all samples for the pair (gene A and B) is then calculated (A vs. B). This calculation is also performed for all the pairs containing gene A such as A vs. C and A vs. D. The arithmetic means of the all SD (mean SD) for gene A represents the variation in the expression of gene A with respect to all the candidate genes, allowing it to be compared with other genes. Accordingly, the gene with the lowest value is the most stable reference gene. A characteristic of this method is that assessments can be performed by merely repeating a relatively simple calculation.

#### BestKeeper

The BestKeeper algorithm of Pfaffl et al.^32^ is characterized by directly inputting the crossing point (CP) value, which is equal to Ct, and PCR efficiency for all the samples of each candidate reference gene to automatically calculate two indicators. The first indicator is the SD of the CP values of each gene, with the lowest value being the most stable reference gene. The developers recommend removing any candidate gene with SD > 1. Next, the geometric mean of the CP values of all genes in each sample is calculated as the BestKeeper index, and Pearson's correlation coefficient (r) between this and the CP value of each gene is measured as the second indicator. The highest value represents the most stable reference gene. Since BestKeeper requires an evaluation of both SD and r, we considered the geometric mean of the ranking for both indicators, which was used to create a general ranking for BestKeeper.

#### NormFinder

The NormFinder algorithm of Andersen et al.^33^ is characterized by evaluating stability through the amount of change in expression within and between groups. A “stability value” is obtained by inputting the relative expression value, with the lowest value representing the most stable reference gene. Each group needs at least eight samples for appropriate assessments and a sample of five to ten candidate reference genes is recommended.

#### geNorm

The geNorm algorithm of Vandesompele et al.^24^ is based on pairwise comparisons. Inputting the relative expression values yields two indicators. The “M value” indicates the stability of expression, with the lowest value representing the most stable reference gene. In the developer’s experimental data, genes with stable expression had a mean M value < 0.5^34^. When evaluating the stability of two or more reference gene combinations, a pairwise variation (V_n/n+1_) is calculated to determine the appropriate number of reference genes to be combined, and V_n/n+1_ < 0.15 is considered as an appropriate cutoff value.

### Statistical analysis

The expression of each gene is presented as mean ± standard deviation (SD). When a significant difference was observed in a one-way analysis of variance (ANOVA) with day as the factor, multiple comparisons with the Bonferroni method were performed. *P* < 0.05 was considered as statistically significant. SPSS 22.0 J for Windows (SPSS Inc., Chicago, IL, USA) was the statistical software used for this analysis.

## Supplementary information

Supplementary file 1 (DOCX 365 kb)
