# Supplementary material for: RT-qPCR analyses on the osteogenic differentiation from human iPS cells: an investigation of reference genes
Source: Sci Rep. 2020 Jul 16;10:11748. doi: 10.1038/s41598-020-68752-2 (PMC7367276; doi:10.1038/s41598-020-68752-2)
Supplement: Supplementary file 1 — (DOCX 365 kb) [file 41598_2020_68752_MOESM1_ESM.docx]

**RT-qPCR analyses on the osteogenic differentiation from human iPS cells: An investigation of reference genes**

Kensuke Okamura^1^, Yusuke Inagaki^1^, Takeshi K. Matsui^2^, Masaya Matsubayashi^2^, Tomoya Komeda^2^, Munehiro Ogawa^1^, Eiichiro Mori^2^, Yasuhito Tanaka^1^

^1^Department of Orthopaedic Surgery, Nara Medical University, Kashihara, Japan

^2^Department of Future Basic Medicine, Nara Medical University, Kashihara, Japan

Correspondence and requests for materials should be addressed to Y. I. (email: yinagaki@naramed-u.ac.jp)

**
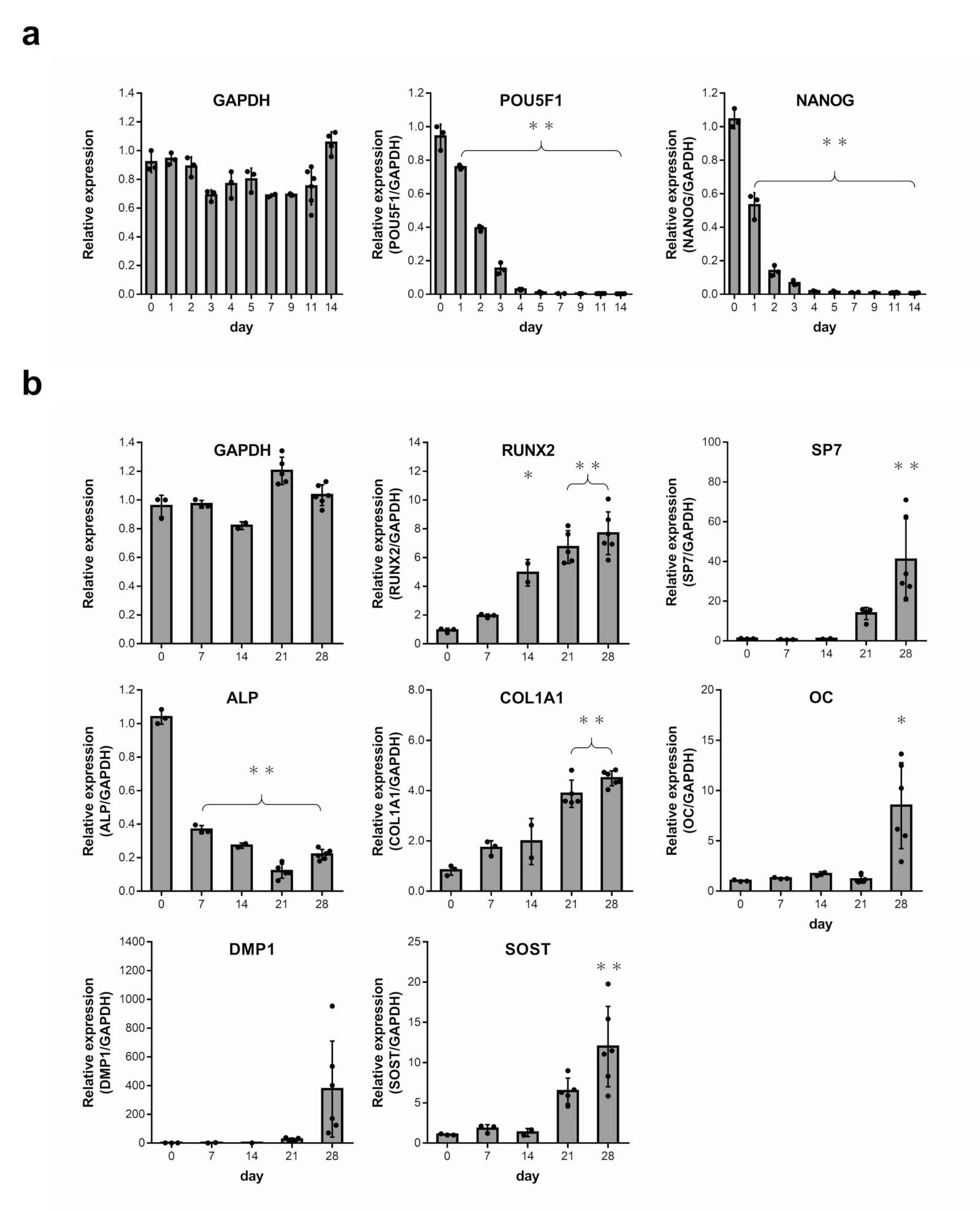
**

**Supplemental Figure 1.** Expression of undifferentiation (a) and osteogenic marker (b) genes standardized by GAPDH in iPSCs during osteogenic differentiation. The values are represented as the mean ± S.D. from two-six independent samples. One-way analysis of variance (ANOVA) with Bonferroni correction for multiple comparisons was applied. *p < 0.05, **p < 0.01 versus day 0.
